# Selenium Biofortification Enhanced Grain Yield and Alleviated the Risk of Arsenic and Cadmium Toxicity in Rice for Human Consumption

**DOI:** 10.3390/toxics11040362

**Published:** 2023-04-11

**Authors:** Fernanda Pollo Paniz, Tatiana Pedron, Vitória Aparecida Procópio, Camila Neves Lange, Bruna Moreira Freire, Bruno Lemos Batista

**Affiliations:** Center for Natural and Human Sciences, Federal University of ABC, Santo André 09210-580, SP, Brazil

**Keywords:** chemical speciation, arsenic, selenium, cadmium, HPLC-ICP-MS, co-exposure, human risk assessment

## Abstract

Arsenic (As) and Cadmium (Cd) are toxic to rice plants. However, selenium (Se) has the potential to regulate As and Cd toxicity. The present study aimed to evaluate the co-exposure to As^5+^ and Se^6+^ species in two rice cultivars, BRS Pampa and EPAGRI 108. The plants were divided into six groups and cultivated until complete maturation of the grains, under greenhouse conditions. Regarding total As and inorganic As (i-As) accumulation in grains, the highest concentrations were found for BRS Pampa. For Se, EPAGRI 108 presented the highest concentration of inorganic and organic Se (i-Se and o-Se). The exposure assessments showed that Se biofortification can mitigate the As accumulation in rice and, consequently, the risk of As and Cd toxicity in grains for human consumption. The combined effect of As and Se in rice plants could represent an alternative to biofortify this food in a safe way and with a higher percentage of bioavailable Se. Although Se is able to mitigate As toxicity in rice plants, in the present study we showed that co-exposure in different cultivars under the same growing conditions may present different responses to As and Se exposure.

## 1. Introduction

Rice is one of the most important foods for human nutrition and is the base diet of more than three billion people. It is the second most cultivated cereal in the world, corresponding to the production of 29% of the total grains used in food [1].

Arsenic (As) is a toxic non-essential semi-metal that can be transferred from plants to food, exposing the population to the risk of poisoning, especially in the long term. Arsenic is found in several chemical forms and oxidation states that can cause adverse chronic and acute health effects, such as cancer [2]. On the other hand, selenium (Se) is also a semi-metal, classified as an essential trace element for the development of human beings at levels of approximately 55 μg day^−1^. Se in elevated doses (>400 µg day^−1^) can be toxic for humans [3,4]. Se and As present chemical similarities, such as the number of shells, electronic structure, and atomic rays. Due to these properties, they can act as antagonists in biological systems [5].

In rice, the uptake of As depends on several factors. Arsenite (As^3+^) or arsenate (As^5+^) from soil are transported into roots by silicate or phosphate transporters, respectively, due to molecular similarities. After this absorption, As^5+^ is converted to As^3+^ by an As-reductase. As^3+^ has good mobility in xylem and phloem, reaching the grains [6]. In addition, As can be methylated by microorganisms, producing dimethyl arsenic (DMA) and monomethyl arsenic (MMA), which cannot be trapped by phytochelatins (PCs) in roots, reaching grains [7]. Se is reported to be a component of many proteins, including glutathione peroxidase (GSH-Px), an antioxidant. Selenoproteins may also protect human and animal systems from heavy metal poisoning [8,9,10]. Wang et al. [9] reported that Se can alleviate lead-caused kidney damage in chickens. In soil and water, Se exists mainly as inorganic ions, as Se^4+^ (selenite) and Se^6+^ (selenate). After being uptaken by roots, the biomethylation process (by micro-organisms and plants) transforms inorganic Se (i-Se) into organic Se (o-Se). Basically, o-Se species are the association of Se and amino acids (selenocysteine-SeCys, selenocysteamine-SeCM, and selenomethionine-SeMet) [11]. Se^6+^, which presents high soil mobility, is transported into roots by sulphate transporters. After absorption, Se^6+^ can be stored in root vacuoles and methylated to SeCys and SeMet [12,13].

There are many studies in the literature about As toxicity in rice cultivars [14,15,16], and some of them are related to As and Se mechanisms in rice plants in the initial phases of development [17,18,19]. However, studies involving As and Se uptake from paddy soil to grain are scarce. Moreover, these studies are short-term cultivation and do not have information about the chemical species of both elements in grains; therefore, the mechanisms involving As and Se uptake in rice grains are poorly understood [18,19,20,21,22,23,24].

In the current study, two indica rice cultivars (BRS Pampa and EPAGRI 108, with different As predilection) were cultivated in soils containing As^5+^, Se^6+^, or the co-exposure of As^5+^ and +Se^6+^ until maturation of grains. Then, we investigated the Se influence on the effects of As (and its chemical species) on the plant agronomic parameters, mitigation of As and Cd accumulated in the grains, and biofortification with organic Se, decreasing the risk to human consumption and improving the nutritional value of the grains.

## 2. Materials and Methods

### 2.1. Reagents and Instruments

Deionized water (resistivity 18.2 MΩ cm, Master System All, Gehaka, Brazil) was used in all experiments. All other reagents used were of analytical grade. Concentrated nitric acid (65% w w^−1^) and NaOH were obtained from Synth (Brazil). As (Na_2_HAsO_4_·7H_2_O), Se (Na_2_SeO_4_) salts and species (dimethyl arsenic—DMA, monomethyl arsenic—MMA, arsenite—As^3+^, and arsenate—As^5+^, selenomethionine—SeMet, selenocystine -SeCys, selenite—Se^4+^, and selenate—Se^6+^) were purchased from Sigma-Aldrich (USA). The analytical standard calibration solutions were from Perkin Elmer (USA). A laboratory oven (SL-100, Solab, Piracicaba, São Paulo) was used for plant drying.

Alpha amylase and protease XIV (Sigma-Aldrich, St. Louis, MO, USA) were used for Se extraction from rice grains. The determination of total concentrations was performed using an inductively coupled plasma mass spectrometer (ICP-MS, Agilent 7900, Hachioji, Japan). High-performance liquid chromatography (HPLC Agilent 1200 Infinity II, Waldbronn, Germany) hyphenated to ICP-MS (HPLC-ICP-MS) was used for As and Se speciation. A digestion block (Easy Digest, Analab, Hoenheim, France) was used for sample preparation. For speciation of 0.22 µm cellulose filters (Sartorius, Burlington, MA, USA), a water bath (SL 1522L, Solab, Brazil), ultrasonic bath (GS9060TH, 3 L, 80 W, 50 KHz, Genesis, New York, NY, USA), orbital shaker (SL 223, Solab, Brazil), and centrifuge (SL 699, Solab, Brazil) were used.

### 2.2. Rice Cultivation and Harvesting

Rice (*Oryza sativa* L.) cultivars EPAGRI 108 and BRS Pampa were used as they were previously classified in another study from our research group as low As accumulator and high As accumulator, respectively. The study involved 30 rice Brazilian cultivars and the data were not published. EPAGRI 108 was developed by the Agricultural Research Corporation of the State of Santa Catarina (Brazil). This cultivar presents high yield potential and grain quality and a late cycle. BRS Pampa was developed in a partnership of the Brazilian Agricultural Research Corporation (EMBRAPA) and Rice Institute of Rio Grande do Sul (Brazil). This cultivar has the characteristic of high genotypic grain yield, adaptability, good agronomic traits, and an early cycle [25,26].

The seeds (approximately 100) were pre-germinated in water for 48 h; then, 3 seeds were planted in 0.2 L-pots (five 0.2 L-pots per treatment). When they were 10 cm long, the seedlings (with soil) were gently removed from the 0.2 L pots and individually transferred to 3.4 L-pots (five 3.4 L-pots per treatment) containing the soil used for cultivation (top soil: organic matter: sand in a 1.5:1.5:1 ratio). The pots of all treatments were randomized disposed int the greenhouse. A total of 15 days after transplanting, the soil was flooded under 3–4 cm of water. The experimental design was composed of 6 treatments with 5 replicates per treatment, as follows (As additions in soils were from 0 to 10 mg L^−1^, based on previous studies [7,15]. Se-additions in soils were 0 or 5 mg L^−1^ to observe the uptake competition at different levels of As):

1. Control: without As^5+^ or Se^6+^ addition in soils;
2. Se5: Se^6+^ at 5 mg L^−1^;
3. As5: As^5+^ at 5 mg L^−1^;
4. As2.5 + Se5: As^5+^ at 2.5 mg L^−1^ and Se^6+^ at 5 mg L^−1^;
5. As5 + Se5: As^5+^ and Se^6+^ at 5 mg L^−1^;
6. As10 + Se5: As^5+^ at 10 mg L^−1^ and Se^6+^ at 5 mg L^−1^.

Plants were grown in a greenhouse with a controlled temperature (~25 °C) and relative humidity (50–60%). The grains of the cultivars EPAGRI 108 and BRS Pampa were collected after complete maturity (248 ± 7 and 269 ± 13 days, respectively). A portion of 200 g of soil from each pot was collected. Finally, the samples (soil and whole grains) were dried in a laboratory oven at 45 °C until constant weight.

### 2.3. Evaluation of Soil and Grain Characterizations

For sample preparation, the soils were homogenized and sieved (<1 mm). Soil texture, pH, organic matter (OM), cationic exchange capacity (CEC), base saturation (%V) K, P, Ca, and Mg were evaluated according to the methodology of Raij et al. [27].

After harvesting, the total number of grains was counted, weighed, and a portion of 20% was measured longitudinally with a caliper rule. The grains were then manually husked.

### 2.4. Determination of As, Cd, and Se in Grains

Total concentrations of As, Cd, and Se were determined in grains. A portion of 150–200 mg of milled samples (<250 µm) were weighed and digested according to Paniz et al. [28]. Standard reference material (NIST 1568b—rice flour) was prepared using the same procedure for all samples.

### 2.5. As Chemical Speciation in Husked Rice

Chemical speciation of As was conducted by HPLC-ICP-MS according to Batista et al. [7]. Approximately 200 mg of husked rice (<250 µm), in triplicate, per treatment was weighed in 50 mL conical tubes. Then, 10 mL of HNO_3_ 2% (v v^−1^) was added and shaken (100 rpm) for 24 h. Subsequently, the tubes were heated at 95 °C in a water bath for 2.5 h and filtered (0.2 µm). The operational conditions of HPLC-ICP-MS are shown in Table S1.

The percentage of As species (%As-sp) in the grains was calculated according to Equation (1):

%As-sp = (C-As-sp × 100)/(T-As-sp) (1)

where %C-As-sp (µg kg^−1^) is the concentration of the As species (As^3+^ or As^5+^ or DMA or MMA), and T-As-sp (µg kg^−1^) is the sum of the concentrations of all As species.

The As species identified in grains were As^3+^, As^5+^, and DMA. The certified reference materials of rice flour (NIST 1568A and European Reference Materials ERM-BC211) were used for quality assurance. The recoveries were 80 and 82.5%, respectively, for organic-As (o-As: MMA + DMA). Recoveries of 92% and 114% were obtained for inorganic-As (i-As: As^3+^ + As^5+^), respectively. A representative chromatogram of ERM rice flour BC-211 is presented in Figure S1A.

### 2.6. The Speciation of Se in Husked Rice

The extraction of Se species was carried out according to Fang et al. [29]. Five mL of deionized water and 25 mg of α-Amylase were added in a 50 mL conical tube containing 250 mg of milled rice (<250 µm). The mixture was kept at a constant temperature of 37 °C in an ultrasonic bath for 30 min and then constantly shaken (100 rpm) for 2 h. Next, 25 mg of Protease XIV was added to the tube, which was kept at 45 °C with constant stirring in an ultrasonic bath for 2 h.

The mixtures were centrifuged at 4500 rpm for 20 min, and the collected supernatants were filtered (0.2 µm) and analyzed by HPLC-ICP-MS (Table S1).

The percentage of Se species (%Se-sp) in the grains was calculated according to Equation (2):

%Se-sp = (C-Se-sp × 100)/(T-Se-sp) (2)

where %C-Se-sp (µg kg^−1^) is the concentration of the Se species (Se^4+^ or Se^6+^ or SeMet or SeCys), and T-Se-sp (µg kg^−1^) is the sum of the concentrations of all Se species. The recoveries of Se species were evaluated by spiking of 25 µg kg^−1^ of each species (Se^4+^, Se^6+^, SeCys and SeMet) in a Control sample. A representative chromatogram of a spiked sample is presented in Figure S2A.

### 2.7. Estimated Daily Intake (EDI)

The estimated daily intake (EDI, µg kg^−1^ BW day^−1^) was calculated to assess the effect of Se biofortification on Se intake and on the human exposure to As through rice consumption. The EDI of As (total, o-As, and i-As), Se (total, o-Se, and i-Se), and Cd through ingestion of rice grains of each of the two rice cultivars studied was estimated according to Equation (3):

EDI = C_ce_ × M_rdc_/BW (3)

where C_ce_ (µg kg^−1^) is the mean concentration of each element or species in rice grains, M_rdc_ is the daily consumption of rice in Brazil, assumed to be 72.6 g day^−1^ [30,31], and BW is the average body weight, assumed to be 70 kg for an adult.

### 2.8. Statistics Analysis

Statistical differences between groups were evaluated by one-way ANOVA followed by Duncan’s multiple range test and critical ranges. All tests were performed using SigmaStat^®®^ (v.3.5, Systat Software Inc., San Jose, CA, USA).

## 3. Results and Discussion

### 3.1. Soil Analysis and Agronomic Parameters

Soil characteristics and textural classification were analyzed and are shown in Table S2. A detailed discussion about the soil used for cultivation can be found in the supplementary material. The results showed that sand was the major component of the soil, followed by clay and silt. Therefore, the classification of the soil is sandy clay loam, in agreement with the soils used in other studies involving rice cultivation [32,33,34].

Table 1 presents the parameters of productivity, grain length, grain weight, and husk weight evaluated in the present study. For EPAGRI 108, no statistical differences (*p* < 0.05) were observed in any agronomic parameter between treatments. Comparing both cultivars, BRS Pampa presented the highest number of husked grains. The length of the grains, grain weight, and husk weight were similar (*p* < 0.05) between cultivars (Table 1). For BRS Pampa, the highest number of grains was obtained for the treatment As10 + Se5 (236 grains), and a statistical difference (*p* < 0.05) was observed when comparing this treatment with the Control, Se 5, and As 5 treatments (Table 1). BRS Pampa treated with As5 + Se5 presented increases of 28% and 12% in the number of grains compared with Se5 and As5 treatments, respectively.

We observed that the number of grains was associated with the weight of the husked grains. In general, an increase in husked grain weight was observed in the treatments submitted to the co-exposure of As and Se (Table 1). For BRS Pampa, the co-exposure As2.5 + Se5 demonstrated statistical difference (*p* < 0.05) compared with the Control treatment. The high exposure to As (5 and 10 mg kg^−1^) in the presence of Se increased the weight of the husked grain by 19% (As5 + Se5) and 52% (As10 + Se5), respectively.

Similar to our findings, Liao et al. [35] observed that the addition of Se at 5 mg kg^−1^ did not affect the grain yield in white rice (H-Liangyou 6839). However, Pokhrel et al. [36] evaluated isolated and co-exposure of As and Se in two indica rice cultivars. The authors reported that the grain yield depends on the concentrations of these elements and the type of cultivar. Exposure to 50 mg kg^−1^ of As^3+^ + 4 mg kg^−1^ of Se^6+^ significantly increased the grain yield for the cultivar Lu You Ming Zhan (LYMZ) and decreased the grain yield for the cultivar Ming Hui 63.

### 3.2. As in Grains: Total Concentration and Chemical Speciation

Table 2 presents the concentration of total As (t-As) in the husked grains for the two rice cultivars studied. Total As in BRS Pampa ranged from 84.7 ± 12.4 µg kg^−1^ (Se5) to 417 ± 52.7 µg kg^−1^ (As10 + Se5). For EPAGRI 108, the t-As ranged from 53.7 ± 5.47 µg kg^−1^ (Se5) to 255 ± 60.4 µg kg^−1^ (As10 + Se5). EPAGRI 108 accumulated lower t-As in the grains than BRS Pampa for the treatment As5.

**Figure 1:**
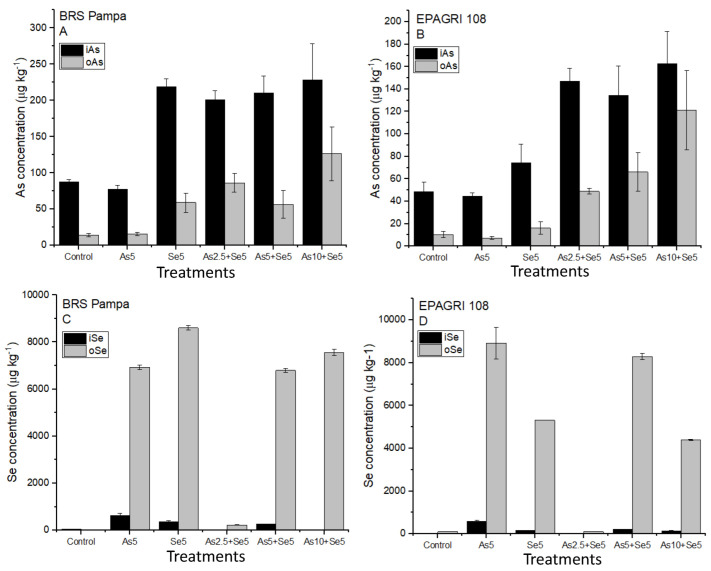
Organic and inorganic As and Se accumulated in the grains of the cultivars tested. Bars not shown are under the limit of detection. Treatments: Control, As5, Se5, As2.5 + Se5, As5 + Se5, and As10 + Se5. (**A**) organic and inorganic As in BRS Pampa cultivar; (**B**) organic and inorganic As in EPAGRI 108 cultivar; (**C**) organic and inorganic Se in BRS Pampa cultivar; (**D**) organic and inorganic Se in EPAGRI 108 cultivar.

Figure 1A,B present the concentrations of As chemical species in the grains. The concentrations of inorganic As (i-As) ranged from 77.3 ± 5.56 µg kg^−1^ (Se5) to 228 ± 50.7 µg kg^−1^ (As10 + Se5) for the cultivar BRS Pampa. For EPAGRI 108, the concentrations of i-As ranged from 44.7 ± 2.97 µg kg^−1^ (Se5) to 163 ± 28.5 µg kg^−1^ (As10 + Se5). No statistical differences (*p* < 0.05) were observed for treatments with similar soil addition of As (Control and Se5, As5, and As5 + Se5). Moreover, the *Codex Alimentarius* recommends i-As levels lower than 350 µg kg^−1^ in husked rice [37] for human intake. Our results demonstrated that all treatments were below this limit, even with the additions of 10 mg kg^−1^ of As in soils (treatment As10 + Se5) for both cultivars.

In terms of As chemical species, the cultivar EPAGRI 108 accumulated lower i-As in the grains than BRS Pampa (Figure 1A,B). As demonstrated previously, t-As concentration in EPAGRI 108 was lower than in BRS Pampa, and this difference may be related to genetic factors and As tolerance.

**Figure 2:**
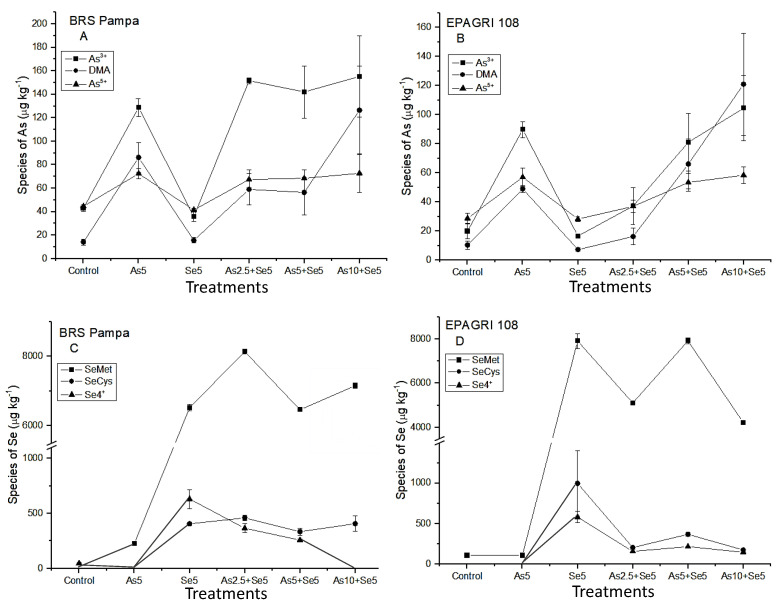
As and Se species in the grains of the cultivars tested. Chemical species and treatments that presented values below LOD: BRS Pampa C: SeMet/SeCys in Control; Se^4+^/SeCys in As5; Se^4+^ in As10 + Se5; EPAGRI 108 D: SeCys/Se^4+^ in Control and As5: (**A**) arsenic species in the grains of BRS Pampa; (**B**) arsenic species in the grains of EPAGRI 108; (**C**) Se species in the grains of BRS Pampa; (**D**) Se species in the grains of EPAGRI 108.

In the present study, As^3+^ was the major As species found in the grains in almost all treatments for both cultivars (Figure 2A,C). For BRS Pampa, the amount of As^3+^ and As^5+^ presented low variations, even with the As increase in soils (treatments As2.5 + Se5; As5 + Se5; and As10 + Se5). For EPAGRI 108, the amount of As^3+^ in the grains was associated with the increasing concentrations of As in the soils. The tolerance of BRS Pampa could be associated with the conversion of As^5+^ by the As-reductase (As^5+^ → As^3+^). This efficient mechanism increased the concentration of As^3+^ that was kept by PCs (in roots and husk), maintaining the concentrations of i-As in the grains constant (Figure 2A). An opposite trend was observed by Syu et al. [15], who studied six rice genotypes (three indica and three japonica, until complete maturation of grains) cultivated under three levels of As in soils (16.3 mg kg^−1^; 343 mg kg^−1^; and 512 mg kg^−1^). The authors observed that DMA was the major As species in the grains, followed by i-As (As^3+^) and the percentage of DMA increased with t-As in rice grains.

Organic forms of As (o-As: DMA) for BRS Pampa ranged from 14.2 ± 2.51 µg kg^−1^ (Control) to 127 ± 37.3 µg kg^−1^ (As10 + Se5). For EPAGRI 108, the o-As ranged from 7.19 ± 1.29 µg kg^−1^ (Se5) to 121 ± 35.3 µg kg^−1^ (As10 + Se5). Similar to the results for i-As, the concentration of o-As also presented significant statistical differences among the treatments with and without soil addition of As for both cultivars (Control and Se5 are statistically different from As5 and As5 + Se5) as reported in Figure 1A,B.

Considering the As chemical species, DMA is the predominant o-As species in grains (Figure 2A,C), corroborating with Norton et al. [38] and Batista et al. [39]. For BRS Pampa, the concentration of DMA was similar in the treatments with the lowest As concentrations (As2.5 + Se5 and As5 + Se5) and 2.2 times higher for As10 + Se5. For EPAGRI 108, this increase was more pronounced (Figure 2C). This can be explained by the high availability of As in the soil that can be methylated by microorganisms, producing DMA and MMA which are not trapped by PCs of the roots, reaching the grains [7].

### 3.3. Se: Total Concentration and Chemical Speciation

The determination of the t-Se in rice grains (Table 2) showed that the soil application of Se^6+^ at 5 mg L^−1^ increased the Se concentrations in rice grains more than 150 times for BRS Pampa and 120 times for EPAGRI 108. Successful biofortification studies with Se application in rice were also performed by Chen et al. [40] and Boldrin et al. [41]. The former tested the foliar application of Se^4+^ and Se^6+^ on a rice cultivar (cv. Teyou 59). Results demonstrated that Se^6+^ application increased SE by 35.9% in grains compared with Se^4+^ application. According to the authors, there were no significant effects of Se application on the crop yield and growth when testing both foliar and soil applications of Se^4+^ and Se^6+^ [40,41]. The authors observed that the application of Se^6+^ in soil was more efficient for Se accumulation in the grains compared with soil addition of Se^4+^.

Some of the literature reports studies involving Se application with the purpose of decreasing the uptake of potentially toxic elements such as As, Pb, and Cd [19,42,43]. Lv et al. [44] evaluated the capacity of Se^4+^ to decrease the uptake of As and Cd in rice plants. The authors used different soil–water regimes to investigate the effects of different doses of Se^4+^ (1 and 5 mg kg^−1^). Their results indicated that the addition of Se stimulated the retention of As and Cd in the Fe/Mn root plaques in soil drying for 10 days. However, the retention was decreased under flooded conditions. The authors also concluded that the rice yield increased during As and Cd exposure, but there were reduced concentrations of many essential elements (Ca, Mn, Fe and Zn) and a higher accumulation of As^3+^ in grains. Finally, they concluded that Se^4+^ was not efficient to decrease As and Cd accumulation in the cultivar YangDao No6. Contrasting with Lv et al. [39], in our study with Se^6+^, the co-exposure of As2.5 + Se5 decreased the Cd uptake in grains by 56% when compared with the Control of BRS Pampa.

The results demonstrated that in both cultivars, the addition of As reduced Cd concentration by 34% and 40% for BRS Pampa and EPAGRI 108, respectively. Anaerobic conditions can increase the mobilization of As in soils due to the increased soil–microbial activity. On the other hand, this soil condition can decrease the mobilization of Cd as it combines with S to form CdS at low redox potential [45].

Figure 1C,D present the concentrations of i-Se and o-Se for BRS Pampa and EPAGRI 108, respectively. We observed that o-Se has a significantly higher concentration than i-Se. Statistical differences (*p* < 0.05) demonstrated that they are similar in the treatments where Se was added. Organic species of Se (o-Se) ranged from <LOD (Control) to 8602 ± 92.9 µg kg^−1^ (As2.5 + Se5) for BRS Pampa; for EPAGRI 108, the concentrations ranged from 106 ± 6.00 (Control) to 8919 ± 740 µg kg^−1^ (Se5) (Figure 1C,D). A representative chromatogram of an Se5 sample (EPAGRI 108) is presented in Figure S2B.

As shown in Figure 2B,D, the i-Se is predominantly in the chemical form of Se^4+^ in both cultivars. Regarding o-Se, SeMet was the major chemical form. These results agree with Li et al. [46], Sun et al. [47], and Carey et al. [48]. These authors reported that o-Se is the predominant form in rice samples, especially in the chemical form of SeMet. The same was observed for wheat, barley, and rye [49].

Selenocysteine is present in lower levels than SeMet (Figure 2B,D). As summarized by Zhu et al. [50], its importance is related to the fact that after i-Se is absorbed by roots, it is transformed into SeCys, and most other organic species are derived from it after enzyme bio-transformations. Therefore, SeCys has the function of being a precursor for bio-synthesis of other o-Se. Carey et al. [48] reported that organic Se (SeMeSeCys and SeMet) increased the concentration of Se in grains by 10–17 times compared to i-Se (Se^4+^ and Se^6+^).

### 3.4. Co-Exposure As and Se: Total Concentration and Chemical Speciation

The t-As in grains was decreased in the presence of Se (*p* < 0.05) for the two rice cultivars studied, as shown in Table 2. When the concentration of As in the soil was duplicated in the presence of Se (As10 + Se5), the concentration of As in the grains was 417 ± 52.7 µg kg^−1^ for BRS Pampa and 255 ± 60.4 µg kg^−1^ for EPAGRI 108. Camara et al. [21] conducted an experiment over 45 days to evaluate the effect of Se (Se^4+^ or Se^6+^) on uptake and translocation of As (As^3+^ or As^5+^) in rice seedlings. After 150 h of exposure, the authors analyzed the roots and shoots. Their results indicated that co-application of As^3+^ and Se^6+^ had little effect on shoots and roots when the exposure was <100 h; however, the presence of Se decreased the levels of As in both parts of the plant during prolonged (150 h) exposure (48.8% and 16.1% in the roots and shoots, respectively). Recently, Pokhrel et al. [36] evaluated the presence of Se^4+^, Se^6+^, As^3+^, and As^5+^ in the soils of two rice cultivars raised until maturation of grains. The authors concluded that the antagonism between As and Se depends on the rice cultivar, As species, and concentration. They also concluded that Se^6+^ had a stronger inhibiting effect than Se^4+^ on the As transfer factor from roots to grains.

In the present study, we also analyzed the grains of co-exposure by chemical speciation (Figure 2). The concentrations of DMA in co-exposure were 59.1 ± 13.3 µg kg^−1^ (As2.5 + Se5) and 56.5 ± 19.3 µg kg^−1^ (As5 + Se5) for BRS Pampa and 16.2 ± 5.55 µg kg^−1^ (As2.5 + Se5) and 66.1 ± 17.2 µg kg^−1^ (As5 + Se5) for EPAGRI 108. These concentrations evidenced that the presence of Se did not influence the concentration of o-As in the grains (mainly as DMA); therefore, the presence of Se was not enough to overcome the main source of DMA in grains: the activity of the microorganisms in the soil [7].

In root cells, As^5+^ is reduced to As^3+^ by an As-reductase. Arsenite can be effluxed to external medium, complexed by thiol peptides (PCs), or translocated to shoots [51]. In the present study, we observed that As^5+^ added in the soils was intensely reduced to As^3+^. For BRS Pampa, the concentrations of As^3+^ had no higher variation between treatments. As related before, this cultivar is more tolerant to As, which explains the higher levels of As^3+^ in all treatments, compared to EPAGRI 108 (Figure 2A,C). The co-existence of Se apparently did not affect the As^3+^ uptake in grains for BRS Pampa. On the other hand, the cultivar EPAGRI 108 had significant variations of As^3+^ between treatments. From treatments As2.5 + Se5 to As5 + Se5, the concentration of As^3+^ is 118% higher, and from treatments As5 + Se5 to As10 + Se5 this difference is 29%. Therefore, the presence of Se minimized As^3+^ uptake in the treatment As10 + Se5.

A study involving As species and Se species absorption in two cultivars until maturation of grains revealed that co-addition of As^5+^ (50 mg kg^−1^) and Se^6+^ (4 mg kg^−1^) in soils decreased As^3+^ in grains 4.76 ± 0.32% for the cultivar MH63 and 15.51 ± 0.53% for the cultivar LYMZ [36]. The addition of Se in soils (0.0, 0.05, and 0.10 mg kg^−1^) with As in the water used for irrigation (0.0, 0.187, 0.375, and 0.75 mg L^−1^) was studied using the cultivar PR-121. The results of grain analysis demonstrated that addition of Se did not affect As uptake by grains [20]. In the present study, comparing As5 and As5 + Se5, the concentrations of As^3+^ in the grains showed that the cultivar BRS Pampa presented an increase of 10.3%, while for EPAGRI 108 the decrease was 9.7%.

The influence of Se species after As addition was evaluated (Figure 2B,D). The chemical species SeMet is predominant. However, the behavior within treatments is the opposite: for BRS Pampa the addition of 2.5 µg kg^−1^ of As in soil increased the amount of SeMet. For EPAGRI 108, the addition of As 2.5 µg kg^−1^ decreased the concentration of SeMet in the grains. When the additions of As were 5 µg kg^−1^ and 10 µg kg^−1^ for BRS Pampa, there was a decrease and increase in the concentration of SeMet in grains, respectively. For EPAGRI 108, an increase and decrease were observed for SeMet in grains, respectively (Figure 2B,D).

Considering the inorganic forms of Se, the increase in As led to decreased levels of Se^4+^ for BRS Pampa; for EPAGRI 108, the levels of Se^4+^ did not change (Figure 2B,D). Selenocysteine remained at the same level for BRS Pampa (334.7–459.9 µg kg^−1^). For EPAGRI 108, SeCys ranged from 174.1 µg kg^−1^ for As10 + Se5 to 368.4 µg kg^−1^ for As5 + Se5. The value of 997.8 µg kg^−1^ was found for Se5, indicating that there is an influence of As addition in soil for EPAGRI 108 cultivar. This same behavior was reported by Han et al. [52]. The authors evaluated the effects of As and Se in Nicotiana tabacum L. The increase in As levels improved the conversion of i-Se →o-Se (SeMet and SeCys). This was associated with the antioxidative effects of Se, where o-Se could directly extinguish reactive oxygen species (ROS), especially hydroxylic free radicals (OH●). This accumulation of ROS in plants is a consequence of stresses from exposure to cold, drought, high light, excess of water, salinity, and heavy metals and metalloids, such as As, Cd, Pb, Al, and Sb [43].

Considering the antagonism of As and Se in rice, Kumar et al. [53] demonstrated in a study of 7 days in rice seedlings, that phosphate application (10 µg mL^−1^) with Se^4+^ (1.5 µg mL^−1^) can decrease the absorption of As^3+^ by roots. Singh, Upadhyay, and Singh [54] observed that the addition of Se^6+^ (2 or 4 mg L^−1^) and As^3+^ (4 mg L^−1^) led to a decrease in As accumulation of 14.24% in root and 23.78% in shoot compared with plants cultivated only with As for 14 days.

### 3.5. The Influence of Single or Combined Exposure to As and Se on the Daily Intake of As, Se, and Cd from Rice

In this study, the exposure to As (total, i-As and o-As), Se (total, i-Se and o-Se), and Cd from consumption of BRS Pampa and EPAGRI 108 rice cultivars was investigated. Table 3 summarizes the EDI results based on the average concentrations obtained for each cultivar and treatment. Considering that for some elements there were significant variations in concentrations, the maximum EDI was also presented, as it could be important from the point of view of human exposure. The regulatory limits for each element and values previously reported for other cultivars and regions are also depicted in Table 3.

Rice biofortification with 5 mg L^−1^ of Se decreased the mean As EDI from BRS Pampa by 18% compared to the Control. The addition of As alone increased the As EDI from BRS Pampa and EPAGRI 108, by 173 and 233%, respectively. Interestingly, combined exposure to As and Se (As5 + Se5) caused a decrease in As EDI compared with single exposure to As (As5) for both cultivars. This decrease was more pronounced for BRS Pampa (20%) than for EPAGR1 108 (10%). In the former, a 33% decrease in o-As EDI was observed, together with a slight increase (5%) in i-As EDI. For EPAGRI, an opposite trend was observed: combined exposure to As and Se decreased i-As EDI by 7%, while increasing o-As EDI by 40% compared with As5. These results confirm the importance of variety selection for biofortification studies, since different cultivars can demonstrate different and even opposite behaviors in relation to element accumulation, especially regarding chemical species. In addition, the results suggest that Se application during rice cultivation in As contaminated environments may be an alternative to reduce As accumulation in grains and, thus, human exposure to As. A study conducted by Chauhan et al. [55] corroborates our findings. The authors found that Se mitigates As toxicity in rice seedlings via reducing As accumulation in hydroponic, short-term experiments.

The As EDI values from rice not treated with this element (Control and Se5) in the present study were similar to the range reported by Freire et al. [30] for Brazilian rice (0.04–0.09 µg kg^−1^ BW day^−1^, from estimated daily intake, in µg per day per person/70, considering a person with a 70 kg body weight). Even for As-treated rice, the maximum EDI found (0.30 µg kg^−1^ BW day^−1^ for the treatment As5 of BRS Pampa) was within the range reported by Kukusamude et al. [56] for Thai rice (0.12–1.05 µg kg^−1^ BW day^−1^, from estimated weekly intake, in μg per kg BW per week/7). Nevertheless, no variety or treatment exceeded the inferior limit (0.3 µg kg^−1^ BW day^−1^) of the benchmark dose lower confidence limit (BMDL) established by the European Food Safety Authority [57,58]. These results indicate that all rice varieties and treatments are safe and have no health hazards for human consumption regarding As exposure.

For Cd, the cultivar BRS Pampa treated with Se (Se5) presented an EDI 44% lower than the Control, which could indicate that Se biofortification may be an effective mechanism to decrease Cd accumulation in rice grains of this cultivar. Differently, for EPAGRI 108, a significant decrease in Cd EDI (33%) was observed for the treatment As5. Considering all treatments, the EDI of Cd in the present study represented 6–25% of the Tolerable Daily Intake calculated from Tolerable Weekly Intake/7) [53]. Therefore, the studied rice cultivars and treatments are safe for human consumption in terms of Cd. Values were within the range reported for Thai rice (0.03–0.13 µg kg^−1^ BW day^−1^) [51] and were higher than those reported for Brazilian rice (0.004–0.009 µg kg^−1^ BW day^−1^) [30].

Finally, biofortification with Se (Se5) caused a sharp increase in Se EDI compared to the Control (157-fold for BRS Pampa and 123-fold for EPAGRI 108). However, in this condition, the Se EDI exceeds the Tolerable Upper Intake Level (UL) from the Institute of Medicine [59] by 37–73%, which is 400 µg daily (5.7 µg kg^−1^ BW day^−1^, considering a person with a 70 kg body weight). Zhang et al. [60] reported a Se EDI of 0.06–1.67 µg kg^−1^ BW day^−1^ (from estimated daily intake, in µg per day per person/70, considering a person with a 70 kg body weight) for Se-rich Chinese rice, an EDI lower than that reported in the present study.

**Table 3:** Mean and maximum (between parenthesis) estimated daily intake (µg kg^−1^ BW day^−1^) of As (total, i-As and o-As), Cd, and Se (total, i-Se and o-Se) through rice consumption.

Cultivar	Treatment	As	i-As	o-As	Se	i-Se	o-Se	Cd
BRS Pampa	Control	0.11 (0.13)	0.09 (0.09)	0.01 (0.02)	0.05 (0.07)	0.05 (0.05)	0.00 (0.00)	0.09 (0.34)
	Se5	0.09 (0.10)	0.08 (0.09)	0.02 (0.02)	7.84 (11.24)	0.65 (0.72)	7.19 (7.25)	0.05 (0.06)
	As5	0.30 (0.36)	0.21 (0.21)	0.09 (0.10)	0.24 (0.49)	0.00 (0.00)	0.24 (0.24)	0.08 (0.14)
	As5 + Se5	0.24 (0.33)	0.22 (0.24)	0.06 (0.08)	7.32 (8.72)	0.27 (0.27)	7.05 (7.06)	0.09 (0.19)
EPAGRI 108	Control	0.06 (0.08)	0.05 (0.06)	0.01 (0.01)	0.08 (0.14)	0.00 (0.00)	0.08 (0.08)	0.03 (0.03)
	Se5	0.06 (0.07)	0.05 (0.05)	0.01 (0.01)	9.85 (12.55)	0.60 (0.65)	9.25 (9.30)	0.03 (0.08)
	As5	0.20 (0.25)	0.15 (0.16)	0.05 (0.05)	0.11 (0.23)	0.00 (0.00)	0.11 (0.11)	0.02 (0.02)
	As5 + Se5	0.18 (0.24)	0.14 (0.15)	0.07 (0.08)	8.71 (12.36)	0.22 (0.22)	8.60 (8.71)	0.03 (0.04)
Reference Intake		0.3–8 ^a^	0.3–8 ^a^		0.79 ^b^–5.7 ^c^			0.36 ^d^
[30] ^e^	Brazilian rice	0.04–0.09	0.03–0.07	0.01–0.02				0.004–0.009
[56] ^f^	Thai rice	0.12–1.05						0.03–0.13
[60] ^e^	Chinese rice				0.05–0.20			
[60] ^e^	Se-rich Chinese rice				0.06–1.67			

^a^ Benchmark dose lower confidence limit (BMDL) (EFSA, 2009); ^b^ from recommended dietary allowance (RDA) (IOM, 2000) of 55 µg daily/70 (considering a 70 kg body weight person); ^c^ from Tolerable Upper Intake Level (UL) (IOM, 2000) of 400 µg daily/70 (considering a 70 kg body weight person); ^d^ from Tolerable Weekly Intake (TWI) (EFSA, 2011) of 2.5 µg per kg BW/7; ^e^ from estimated daily intake (µg per day per person)/70 (considering a 70 kg body weight person); ^f^ from estimated weekly intake (μg per kg BW per week)/7.

The combined application of As and Se (As5 + Se5) resulted in a slight decrease in Se EDI in both cultivars compared with Se5. Although this decrease was noted for both o-Se and i-Se, it was more pronounced for i-Se (58–63%) than for o-Se (2–7%). It is known that there are remarkable species-dependent differences in toxicity and bioavailability of Se, e.g., i-Se species can be more toxic, while o-Se species are in general more bioaccessible [61,62,63]. In fact, Rohn et al. [63] found an inverse relationship between toxicity and bioavailability of Se species, with organic species being less toxic and more bioavailable. In this scenario, the combined effect of As and Se in rice plants may be an alternative to biofortify this food in a safe way and with a higher percentage of bioavailable Se. Furthermore, it would be possible to use this Se-rich rice as a basis to produce other foods fortified with Se, such as biscuits, flours, and beverages, as a strategy to reduce Se deficiency among the population.

## 4. Conclusions

The results of co-exposure of Se and As demonstrated that different cultivars grown under the same conditions until complete maturation of grains may present different responses on the agronomic parameters and on the uptake and accumulation of Se and As. In the current study, the results of t-As in grains demonstrated that BRS Pampa has more tolerance than EPAGRI 108, and EPAGRI 108 accumulates more Se than BRS Pampa. Both cultivars accumulate basically o-Se, with significant increasing concentrations in the grains.

Finally, single or combined exposure to As and Se affected daily intake of As, Se, and Cd for both cultivars. Results indicated that Se biofortification mitigated As and Cd toxicity in rice by reducing the accumulation of these elements in grains, mainly for BRS Pampa. Furthermore, the combined effect of As and Se in rice plants produced grains with a higher percentage of non-toxic, more bioavailable Se species. Due to the difficulty when planting rice, there are few studies in the literature that examine rice cultivation until complete maturation of grains. Therefore, researchers must be encouraged to develop long-term studies to better understand the interactions between As and Se in rice and the risk of its consumption.

## Figures and Tables

**Table 1:** Agronomic parameters of the grains harvested after complete maturation of the cultivars BRS Pampa and EPAGRI 108. Results are expressed as mean ± standard deviation (n = 5); ^a,b,c^: significant statistical differences at *p* < 0.05.

		Parameters Evaluated			
	Treatments	Number of Grains	Length (mm) of Grains	Weight (g) of Grains	Weight (g) of Husk’s Grains
BRS PAMPA	Control	149 ± 49	8.8 ± 0.2	2.7 ± 0.8	0.9 ± 0.4
	Se5	134 ± 22	8.6 ± 0.4	2.6 ± 0.5	0.8 ± 0.1
	As5	154 ± 51	8.7 ± 0.2	2.6 ± 0.8	0.9 ± 0.4
	As2.5 + Se5	184 ± 62 ^bc^	9.1 ± 0.1	3.4 ± 0.9 ^b^	1.0 ± 0.3
	As5 + Se5	172 ± 56 ^b^	8.8 ± 0.2	3.1 ± 1.0	0.9 ± 0.3
	As10 + Se5	236 ± 80 ^abc^	8.7 ± 0.3	4.1 ± 1.5 ^abc^	1.2 ± 0.4
EPAGRI 108	Control	108 ± 30	9.3 ± 0.1	2.6 ± 0.6	0.8 ± 0.2
	Se5	124 ± 31	9.2 ± 0.2	2.7 ± 0.7	0.8 ± 0.2
	As5	108 ± 17	9.2 ± 0.1	2.4 ± 0.4	0.7 ± 0.1
	As2.5 + Se5	125 ± 49	9.3 ± 0.2	2.8 ± 1.1	0.7 ± 0.3
	As5 + Se5	173 ± 43	9.1 ± 0.3	3.6 ± 1.0	1.0 ± 0.3
	As10 + Se5	132 ± 52	9.1 ± 0.2	3.0 ± 1.2	0.8 ± 0.3

^a^ Statistical difference between Control and evaluated treatments; ^b^: statistical differences between treatments with Se5 or As5; ^c^: statistical differences between treatments with Se5 or As5 and co-exposure (As2.5 + Se5; As5 + Se5; or As10 + Se5).

**Table 2:** Concentration of As, Se, and Cd in grains of the cultivars BRS Pampa and EPAGRI 108 in all treatments. Results expressed as mean ± standard deviation (n = 5); ^a,b,c^: significative statistical differences at *p* < 0.05.

Treatment	Cultivar: BRS Pampa (Mean ± Standard Deviation)			Cultivar: EPAGRI 108 (Mean ± Standard Deviation)		
	As (µg kg^−1^)	Se (µg kg^−1^)	Cd (µg kg^−1^)	As (µg kg^−1^)	Se (µg kg^−1^)	Cd (µg kg^−1^)
Control	107 ± 10	48 ± 11	83.0 ± 24.5	61 ± 14	79 ± 32	27.8 ± 2.81
Se5	85 ± 12	7560 ± 2029 ^a^	49.2 ± 4.58 ^a^	54 ± 6	9499 ± 1801 ^a^	28.9 ± 6.38
As5	293 ± 34 ^ab^	227 ± 146 ^b^	75.0 ± 34.1	197 ± 34 ^a^	107 ± 67 ^ab^	16.7 ± 1.31
As2.5 + Se5	263 ± 25 ^ab^	8968 ± 1864 ^a^	42.4 ± 26.4 ^a^	94 ± 17 ^ab^	5474 ± 949 ^ab^	43.3 ± 17.0
As5 + Se5	229 ± 21 ^ab^	7055 ± 1047 ^ac^	91.4 ± 30.7	170 ± 48 ^ab^	8400 ± 3372 ^a^	32.6 ± 11.2
As10 + Se5	417 ± 53 ^abc^	7565 ± 1154 ^ac^	63.4 ± 3.51	255 ± 60 ^abc^	4543 ± 21 ^abc^	37.0 ± 10.2

^a^: Statistical differences between Control and evaluated treatments; ^b^: statistical differences between treatments Se5 or As5; ^c^: statistical differences between treatments Se5 or As5 and co-exposure (As2.5 + Se5; As5 + Se5; or As10 + Se5).

## Data Availability

All data generated or analyzed during this study are with the corresponding author, and, if necessary, he is available to answer any questions about the datasets, which can be requested by reasonable request.

## Supplementary Materials

The following supporting information can be downloaded at: https://www.mdpi.com/article/10.3390/toxics11040362/s1, Figure S1: Representative chromatogram of typical As speciation by HPLC-ICP-MS. ERM Rice Flour BC 211 (A) and rice sample of the treatments As10 + Se5 (BRS Pampa, (B)). Table S1 shows the operational conditions used for analysis; Figure S2: Representative chromatogram of typical Se speciation by HPLC-ICP-MS. Spiked (25 µg L^−1^) rice sample (A) and rice sample Se5 (EPAGRI 108, (B)). Table S1 shows the operational conditions used for analysis; Table S1: Instrumental operating conditions for determination of the total concentrations of Se, As, and chemical speciation; Table S2: Characteristics of the soil used for rice cultivation. Results expressed as mean (n = 1 per treatment). Note: The soils used in all treatments were prepared on the same day and under the same conditions. References [64,65,66,67,68,69] are cited in the supplementary materials.
