# Supplementary material for: Selenium Biofortification Enhanced Grain Yield and Alleviated the Risk of Arsenic and Cadmium Toxicity in Rice for Human Consumption
Source: Toxics. 2023 Apr 11;11(4):362. doi: 10.3390/toxics11040362 (PMC10143363; doi:10.3390/toxics11040362)
Supplement: Supplementary file 1 [file toxics-11-00362-s001.zip › toxics-2226347-supplementary.pdf]

Supplementary Material

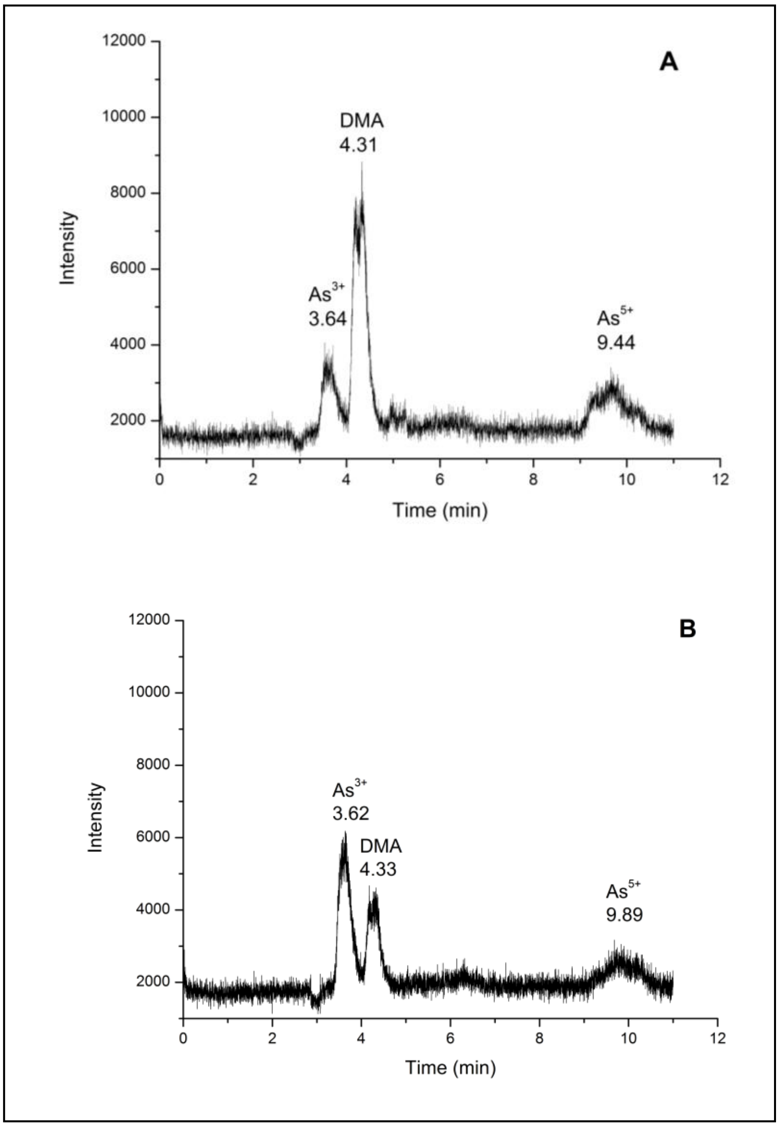

Figure S1. Representative chromatogram of typical As speciation by HPLC-ICP-MS. ERM Rice Flour BC 211 (**A**) and rice sample of the treatment As10+Se5 (BRS Pampa, **B**). Table S1 shows the operational conditions used for analysis.

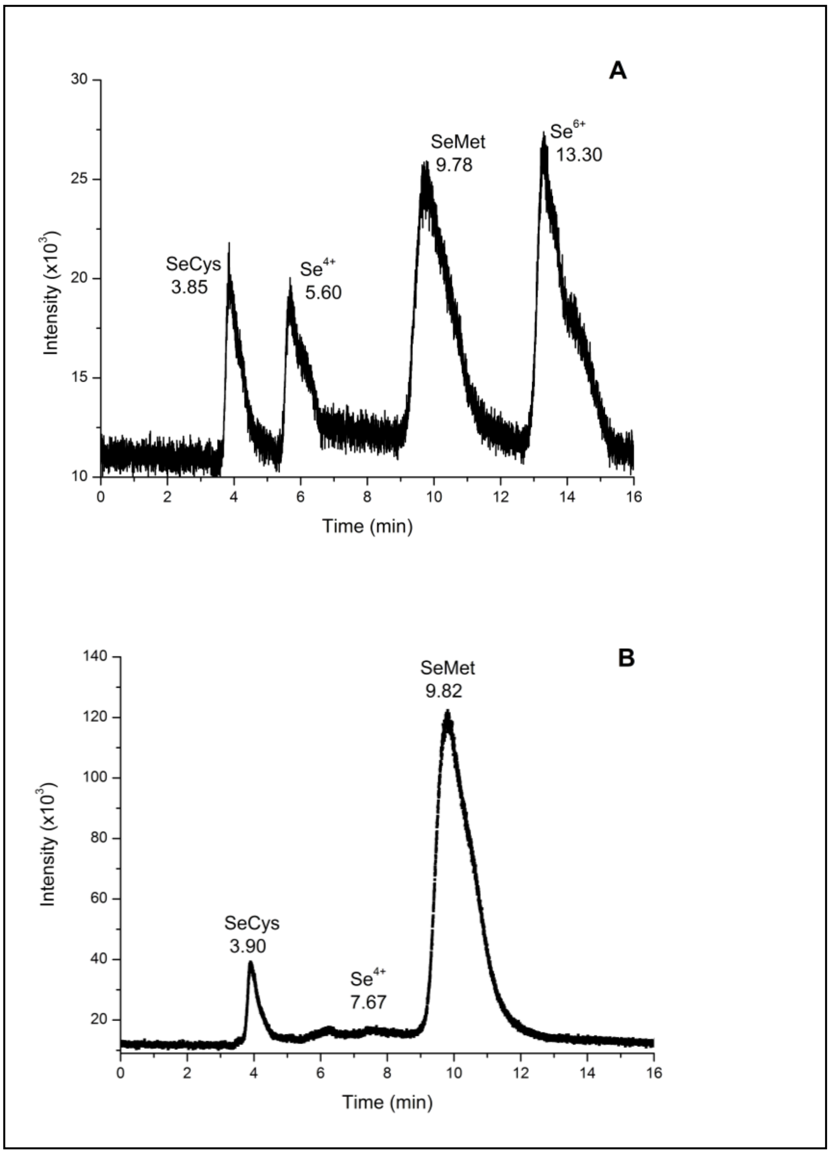

Figure S2. Representative chromatogram of typical Se speciation by HPLC-ICP-MS. Spiked (25 μg L^{-1}) rice sample (**A**) and rice sample Se5 (EPAGRI 108, **B**). Table S1 shows the operational conditions used for analysis.

Table S1. Instrumental operating conditions for determination of the total concentrations of Se and As and chemical speciation.

**ICP-MS: Operating conditions**	
Monitored isotopes and LOD (μg kg^{-1})	^{75}As (0.01), ^{78}Se (0.59)
Radio Frequency Power	1550 W
Torch sample depth	8 mm
Argon Flow rate	15 L min^{-1}
Nebulizer gas flow rate	0.9 L min^{-1}
Nebulizer	Micro Mist™
Sample uptake	0.45 rps
Sample introduction	1.0 mL min^{-1}
Carrier nebulizer gas	1.03 L min^{-1}
Flow rate stabilize time	5 s
Collision cell	He (purity>99.99%)
Nebulizer chamber	Scott (double pass)
Interface cones	Platinum cones
Sampler	0.9 mm
Skimmer	0.45 mm
**HPLC: Operating conditions**	
**As Speciation**	
LOD (μg kg^{-1})	As^{3+} (3.5), As^{5+} (20.5), DMA (5.0)
Column (ion exchange)	Hamilton PRP X-100 (150mm x 4.6mm, 5μm)
Composition of the mobile phase	98% v v^{-1} 10 mM Ammonium phosphate buffer 2% v v^{-1} Methanol, pH 8.2
Mobile phase flow	1.0 mL min^{-1}
Column temperature	25^0C
Run time	11 min
Mode	Isocratic
Injection volume	40 μL
Measurement	Peak area
**Se Speciation**	
LOD (μg kg^{-1})	Se^{4+}(30), Se^{6+} (35), SeCys (39), SeMet (80)
Column (ion exchange)	Hamilton PRP X-100 (150mm x 4.6mm, 5μm)
Composition of the mobile phase	99% v v^{-1} 10 mM Ammonium citrate buffer 1% v v^{-1} Methanol, pH 5.0
Mobile phase flow	0.4 mL min^{-1}
Column temperature	25 ^0C
Run time	16 min
Mode	Isocratic
Injection volume	20 μL
Measurement	Peak area

Table S2. Characteristics of the soil used for rice cultivation. Results expressed as mean (n=1 per treatment). Note: The soils used in all treatments were prepared in the same day and conditions, therefore, the determinations of all treatments are from the same soil.

Treatments	Soil characteristics							
	OM[a] (g dm^{-3})	CEC[b]	Sand (g kg^{-1}) coarse	fine	total	Clay (g kg^{-1})	Silt (g kg^{-1})	Classification
**Control**	30	105	302	283	585	218	197	SLC[f]
**Se5**	27	88	321	235	556	237	207	SLC[f]
**As5**	29	98	332	270	602	204	194	SLC[f]
**As2.5+Se5**	29	97	329	289	618	240	142	SLC[f]
**As5+Se5**	28	102	331	246	577	245	178	SLC[f]
**As10+Se5**	28	87	329	326	655	206	139	SLC[f]
**Control**	29	83	268	259	527	229	244	SLC[f]
**Se5**	27	77	301	252	553	211	236	SLC[f]
**As5**	33	91	288	295	583	225	192	SLC[f]
**As2.5+Se5**	31	80	266	304	570	225	205	SLC[f]
**As5+Se5**	29	73	323	290	613	200	187	SLC[f]
**As10+Se5**	31	77	320	348	668	206	126	SLC[f]

[a] Organic matter; [b] Cationic exchange capacity.

Detailed discussion of the soil used for cultivation

The macronutrients are essential for plant development. For BRS Pampa the concentration of Ca, Mg, K e P were 70 ± 7 mmolc dm^{-3}; 12 ± 1,5 mmolc dm^{-3}; 4,9 ± 0,3 mmolc dm^{-3} and 17 ± 1,3 mg dm^{-3}, respectively for all treatments. For EPAGRI 108 the values of Mg, K e P were similar to BRS Pampa. Calcium presented the value of 54 ± 6 mmolc dm^{-3}.

The cationic exchange capacity (CEC) represents the capacity of soil to release nutrients keeping the fertility of soil for an extend period (Ronquim, 2010). Our values of CEC were 88 to 105 mmolc dm^{-3} for BRS Pampa and from 73 to 91 mmolc dm^{-3} for EPAGRI 108 (Table S2). The pH ranged from 6.5 to 7.5 for both cultivars. The pH was considered normal once the pH in flooded soils is approximately 7 (Ponnamperuma, 1972). The CEC (ranging from 50 to 150 mmolc dm^{-3}) is considered an important parameter for plant development (Sobral *et al.*, 2015). The levels of base saturation (%V) are above 80% for all treatments and the soils can be considered eutrophic (%V >50) (dos Santos *et al.*, 2018). Organic matter (OM) is related with the soil's texture and values from 16-30 g dm^{-3} are related to medium texture of soil (good equilibrium with sand, silt

and clay) (IAL, 2020). In the present study, the concentrations of OM were considered intermediary (mean of 29.5 g dm^{-3}, Table S2). Therefore, all soil characteristics were similar between the treatments.

Soils with different characteristic of texture is not a limiting to rice cultivation. Da Silva *et al.*, (2020) evaluated the levels of As, Cd, Co, Cu, Fe, Mg, Mn, Ni, Pb, Se and Zn in husked grains cultivated under different soil water tensions in different phases of development in three rice cultivars, including BRS Pampa. The texture of their soil was loam, with pH >4.3, OM ranged from 12-17 g dm^{-3} and CEC's values are from 72 to 84 mmolc dm^{-3}. According to authors, the characteristics of soil were similar among of three years of study.

References

RONQUIM, Carlos César. Conceitos de fertilidade do solo e manejo adequado para as regiões tropicais. Embrapa Monitoramento por Satélite. **Boletim de Pesquisa e Desenvolvimento**, 2010.

Ponnamperuma, F. N. (1972). The chemistry of submerged soils. In **Advances in agronomy** (Vol. 24, pp. 29-96). Academic Press.

SOBRAL, L. F., BARRETO, M. D. V., Da Silva, A. J., & Dos Anjos, J. L. (2015). Guia prático para interpretação de resultados de análises de solos. ***Embrapa Tabuleiros Costeiros-Documentos*** *(INFOTECA-E)*.

dos Santos, H. G., JACOMINE, P. K. T., Dos Anjos, L. H. C., De Oliveira, V. A., LUMBRERAS, J. F., COELHO, M. R., ... & CUNHA, T. J. F. (2018). ***Sistema brasileiro de classificação de solos*****.** Brasília, DF: Embrapa, 2018..

IAL-Instituto Agronômico de Campinas (iac.sp.gov.br), http://www.iac.sp.gov.br/produtoseservicos/analisedosolo/interpretacaoanalise.php, accessed on 05/05/2021.

da Silva, J. T., Paniz, F. P., Pedron, T., Torres, D. P., da Rocha Concenço, F. I. G., Parfitt, J. M. B., & Batista, B. L. (2020). Selected soil water tensions at phenological phases and mineral content of trace elements in rice grains–mitigating arsenic by water management. ***Agricultural Water Management*****,** *228*, 105884.
